# High genetic diversity among *Mycobacterium tuberculosis *complex strains from Sierra Leone

**DOI:** 10.1186/1471-2180-8-103

**Published:** 2008-06-25

**Authors:** Susanne Homolka, Erik Post, Barbara Oberhauser, Abu Garawani George, Lars Westman, Foday Dafae, Sabine Rüsch-Gerdes, Stefan Niemann

**Affiliations:** 1 National Reference Center for Mycobacteria, Research Center Borstel, Borstel, Germany; 2 German Leprosy and TB Relief Association, Würzburg, Germany; 3 National Leprosy/TB Reference Laboratory, Freetown, Sierra Leone; 4 National Program Manager for Tuberculosis and Leprosy, Freetown, Sierra Leone

## Abstract

**Background:**

Among tuberculosis (TB) high incidence regions, Sub-Saharan Africa is particularly affected with approx. 1.6 million new cases every year. Besides this dramatic situation, data on the diversity of *Mycobacterium tuberculosis *complex (MTBC) strains causing this epidemic in this area are only sparsely available. Here we analyzed the population structure of strains from Sierra Leone with a special focus on the prevalence of *M. africanum*.

**Results:**

A total of 97 strains isolated from smear positive cases registered for re-treatment in the Western Area and Kenema districts in years 2003/2004 were investigated by susceptibility testing (first line drugs) and molecular typing (IS*6110 *fingerprinting, spoligotyping, and MIRU-VNTR typing).

Among the strains analyzed, 32 were resistant to isoniazid, and 11 were multidrug resistant (at least resistant to isoniazid and rifampin). The population diversity was high with two previously described *M. africanum *lineages (West African-1, n = 6; West African-2, n = 17) and seven *M. tuberculosis *lineages (Haarlem, n = 14; LAM, n = 15; EAI, n = 4; Beijing, n = 4; S-type, n = 4, X-type, n = 1; Cameroon, n = 4). Furthermore, two new *M. tuberculosis *genotypes Sierra Leone-1 (n = 7) and -2 (n = 10) were found. Strain classification according to a 7 bp deletion in pks1/15 revealed that the majority of *M. tuberculosis *strains belonged to the Euro American lineage (66 out of 74).

**Conclusion:**

Resistance rates in Sierra Leone have reached an alarming level. The population structure of MTBC strains shows an intriguing diversity raising the question of possible consequences for TB epidemic and for the introduction of new diagnostic tests or treatment strategies in West Africa.

## Background

Pathogens of the *Mycobacterium tuberculosis *complex (MTBC) such as *M. tuberculosis *and *M. africanum *are the causative agents of tuberculosis (TB) and remain one of the leading infectious killers worldwide. According to the latest WHO report, there have been approx. 8.8 million new TB-cases worldwide in year 2005 and 1.6 million people died of TB [1]. Furthermore, treatment and control of TB is increasingly complicated by the emergence of drug resistant or even multidrug resistant (MDR, resistance to at least isoniazid [INH] and rifampin [RMP]) strains [2]. Levels of MDR-TB among new patients have reached 14% in some areas of the world making successful treatment very difficult [2]. Among the regions with a high incidence of TB, Sub Saharan Africa is particularly affected with approximately 2 million new cases every year [1,3]. Furthermore, the TB epidemic in Africa is reinforced by the mutual interaction of TB and HIV as HIV increases the risk of reactivation of latent TB as well as the rapid progression to active TB after infection [4].

Besides this dramatic situation in several Sub Saharan countries, data on the diversity of MTBC strains causing the epidemic in this area are only sparsely available. However, recent reports indicate that the genetic heterogeneity of MTBC strains is higher than previously anticipated and may influence host pathogen interaction, immunogenicity, transmissibility, development of drug resistance, and the performance of diagnostic tests [5-8]. Starting with the introduction of the first effective strain genotyping technique, IS*6110 *DNA fingerprinting [9], a number of further molecular methods such as spoligotyping [10], investigation of chromosomal deletions (regions of difference, RD), and most recently mycobacterial interspersed repetitive unit-variable number of tandem repeats (MIRU-VNTR) analysis have been established to analyze the diversity and population structure of clinical MTBC strains [6,10-12]. These studies confirmed a phylogeographical population structure of the MTBC. In addition to the classical species *M. tuberculosis, M. africanum, M. microti, M. caprae, M. bovis *and *M. prototuberculosis *(including *M. canetti*), several well defined sub lineages were described within every species such as Haarlem or Beijing genotype among *M. tuberculosis *and West African-1 and West African-2 among *M. africanum *[6,11,13]. Based on the new tools for the efficient analysis of the MTBC population structure, an increasing number of investigations mounted evidence that the bacterial genomic background indeed can influence disease related characteristics [8,14]. This might result e.g. in an enhanced capability to spread or an enhanced propensity to gain drug resistance when certain genotypes were considered and can influence diagnostic test such as the newly developed interferon-gamma-based T cell assays [5,8,14].

In contrast to Europe and USA, where the majority of human TB cases is caused by *M. tuberculosis*, a significant number of TB cases in West Africa has been reported to be caused by *M. africanum *strains which can be further subdivided into the two main lineages West African I and West African II [6,15]. While some studies suggest a lower virulence and infection frequency of *M. africanum *which might result in a longitudinal extinction of this species [6,16,17], others still indicated higher levels of *M. africanum *infection prevalence and did not find significant differences in clinical presentation between *M. africanum *and *M. tuberculosis *[18,19]. It appears to be a geographical gradient in the distribution of both *M. africanum *lineages with West African-1 dominating in Cameroon and West African-2 dominating in Gambia. However only a limited amount of data based on molecular classification of clinical isolates is available.

To further investigate this question, we have performed a retrospective study on the population structure of clinical MTBC strains obtained from patients registered for re-treatment in Sierra Leone in years 2003/2004 with a special focus on the prevalence of *M. africanum*. Sierra Leone is one of the high burden countries in West Africa with a TB incidence of 475/100.000 and TB mortality of 111/100.000 in year 2005 [1]. A study from 1993 reported a significant number of *M. africanum *infections in that region and was used to investigate the longitudinal trend of *M. africanum *prevalence [20]. In addition to molecular typing, drug susceptibility testing was carried out to monitor the level of MDR-TB among previously treated patients in the study area.

## Results and Discussion

In the study period, MTBC cultures were grown from specimens of 103 patients registered for re-treatment between March 2003 and June 2004 in the Western Area and Kenema districts in Sierra Leone. Chromosomal DNA was successfully isolated from 97 strains which were used for further analysis. Six strains were not viable at the time when the DNA isolation was carried out. The main reason for registration for re-treatment among the 97 patient were relapse (n = 41) and treatment after interruption (n = 38). Twelve patients were treatment failure and for six no data are available.

The level of drug resistance was high; resistance to any first line drug was found in 50 patients (52%), any resistance to INH was documented in 32 patients (33%), 14 were resistant to RMP (14%), and MDR-TB was found in 11 patients (11%). The detailed distribution of drug resistance is shown in Table 1. Our data demonstrate that drug resistance rates in re-treatment case in Sierra Leone have reached a level that is comparable with that of other resistance "hot spots" e.g. located in Eastern Europe [2,21]. These findings argue strongly for a systematic surveillance of drug resistance rates in new as well as previously treated cases to get a clear picture on the actual resistance situation in Sierra Leone. Systematic susceptibility testing to rapidly detect drug resistance should be implemented in combination with effective treatment adapted to individual resistance profiles. This is essential to avoid an ongoing spread of resistant and MDR strains especially in a particularly vulnerable population with a high HIV rate. The potential danger posed by such strains has recently been shown by the longitudinal spread of a highly transmissible MDR strain in the KwaZulu-Natal region which has finally resulted in the development of extensively drug resistant variants [22].

**Table 1:** Resistance to first line antituberculosis drugs among the 97 strains investigated.

	No. (%)
Total tested	97
Fully sensitive	47 (48.5)
Any resistance	50 (51.5)
- Any S resistance	40 (41.2)
- Any H resistance	32 (33.1)
- Any R resistance	14 (14.4)
- Any E resistance	15 (15.5)
- Any Z resistance	10 (10.3)
Any Mono-resistance	24 (24.7)
- S only	15 (15.5)
- H only	8 (8.2)
- R only	1 (1,0)
- E only	-
- Z only	-
H and R resistance	
- MDR	11 (11.3)
Other patterns	12 (12.4)
- S + H only	4 (4.1)
- S + H + E only	4 (4.1)
S + H + Z only	2 (2.1)
S + H + E + Z	2 (2.1)

Abbreviations: H, isoniazid; R, rifampicin; E, ethambutol, Z, pyrazinamide; S, streptomycin.

To investigate the population structure of the 97 MTBC strains, molecular typing with IS*6110 *DNA fingerprinting, spoligotyping and analysis of 24 MIRU-VNTR loci was performed. For all strains, clear cut IS*6110 *DNA fingerprint and spoligotyping profiles were obtained. MIRU-VNTR typing was also successful for all strains with the exception that VNTR 2163b could not be amplified for one strain and an exceptional large fragment was obtained for VNTR 0577 for one isolate.

**Figure 1:**
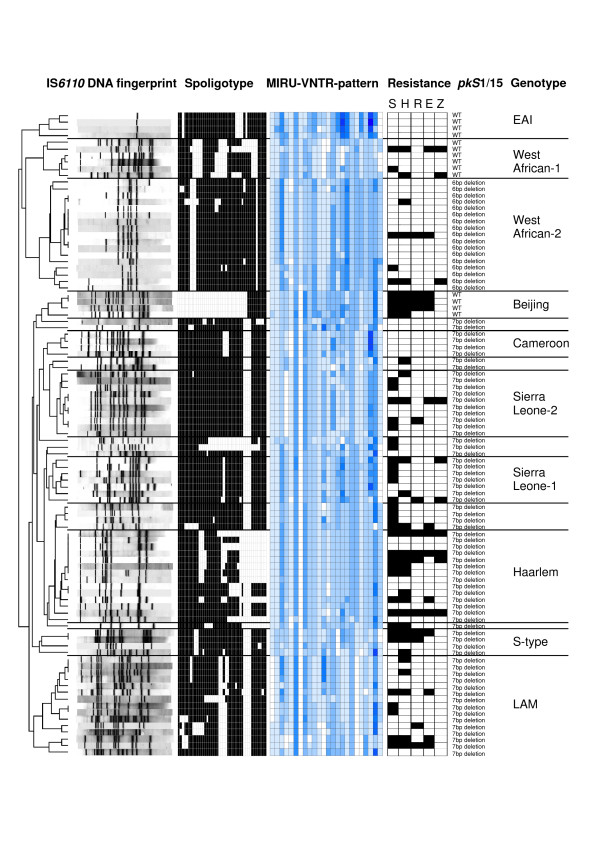
**IS*6110 *DNA fingerprint, spoligotype and 24 loci MIRU-VNTR typing patterns of the 97 strains investigated**. The position of each IS*6110 *band is normalized, so that banding patterns of all strains are mutually comparable. The copy numbers of 24 MIRU loci (see Materials and Methods section) are displayed in grey shades ranging from 0 (white) to 14 copies (black). The strains genotypes are ordered in a dendogram based on the similarity of their MIRU-VNTR typing data. In addition resistance to first line drugs is shown by a black box. Abbreviations: WT, wild type; EAI, *M. tuberculosis *East African Indian; LAM, *M. tuberculosis *Latin American Mediterranean.

All genotyping data were digitized and analyzed with the Bionumerics software. A dendrogram based on the similarity of MIRU-VNTR typing data revealed a surprising high population diversity comprising several groups which can be mainly allocated to MTBC genotypes described previously (Fig. 1; for MIRU copy numbers and SpolDB4 types see Additional file 1). Genotype identification was carried out according to SpolDB4 [13] and by using the freely assessable MIRU-VNTR*plus *web database [23], which comprises genotyping data of a reference strain collection of validly described MTBC genotypes.

Overall, 23 strains (24%) were identified as *M. africanum *and 74 as *M. tuberculosis *(76%). The most prominent genotype was *M. africanum *West African-2 (17.5%) followed by *M. tuberculosis *LAM (15.5%) and Haarlem (14.4%) (Table 2). In addition to genotypes described before, we identified two new strain types which are defined by closely related IS*6110 *DNA fingerprint and MIRU-VNTR patterns and were designated Sierra Leone-1, and Sierra Leone-2, respectively (Fig. 1). In total, this accounts to a great variety of a total of 11 different genotypes/phylogenetic lineages that were present among this small sample of strains from Sierra Leone (Table 2).

**Table 2:** Classification of the 97 strains analyzed in *M. tuberculosis *complex species and genotypes

**Classification**	**No. (% of all)**	**pks 1/15 (%)**	**No. MDR (%)^1^**
*M. tuberculosis*			
Total	74 (76.3)		10 (13.5)
Beijing	4 (4.1)	WT (100)	3 (75%)
Cameroon	4 (4.1)	7 bp deletion (100)	0
East African Indian	4 (4.1)	WT (100)	0
Haarlem	14 (14.4)	7 bp deletion (100)	4 (28.6%)
LAM	15 (15.5)	7 bp deletion (100)	1 (6.7)
Sierra Leone-1	7 (7.2)	7 bp deletion (100)	0
Sierra Leone-2	10 (10.3)	7 bp deletion (100)	0
S-type	4 (4.1)	7 bp deletion (100)	2 (50%)
X-type	1 (1.0)	7 bp deletion (100)	0
No classification	11 (11.3)	7 bp deletion (100)	
*M. africanum*			
Total	23 (23.7)		1 (4.3)
West African-1	6 (6.2)	WT	0
West African-2	17 (17.5)	6 bp deletion (100)	1 (5.9)
Total	97 (100)		11 (11.3)

^1^% of strains of the respective genotype

Abbreviations: WT, wild type; bp, base pair.

**Figure 2:**
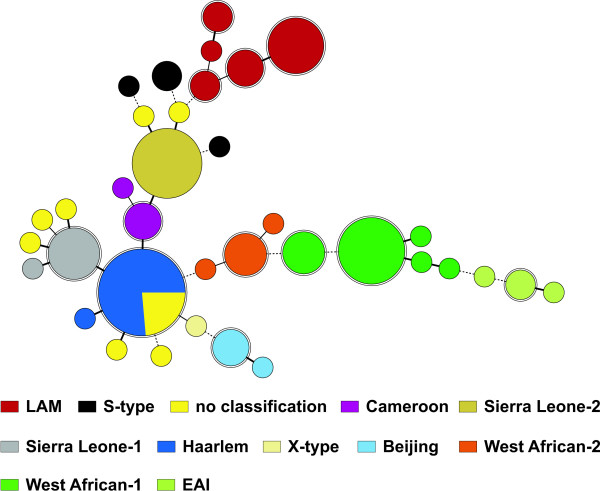
**Minimum spanning tree based on the diversity of MIRU-VNTR data**. Circles show the different clonal complexes identified (maximum neighbour distance: 4 changes; minimum size: 2 MIRU-VNTR types) by the set of 24 loci among the 97 MTBC strains analyzed. The size of each circle is proportional with the number of MIRU-VNTR types belonging to a particular complex. Colours indicate strain previous classification (Table 2). EAI, *M. tuberculosis *East African Indian; LAM, *M. tuberculosis *Latin American Mediterranean.

To further confirm the genotype classification, we used the MIRU-VNTR data to calculate a Minimum Spanning Tree (MST), which uses a maximum parsimony algorithm to investigate phylogenetic relationships and to identify clonal complexes within a population. The MST strikingly confirmed the genotype classification according to UPGMA tree based analysis (Fig. 1) and to comparison with the MIRU-VNTR*plus *web database [23]. All lineages suspected from dendrogram-based analysis were also detected as clonal complexes in the MST (Fig. 2) including the newly described genotypes Sierra Leone-1 and -2, thus confirming the existence of previously undefined phylogenetic lineages in Sierra Leone. The MST analysis also helped to assign strains with unclear classification to known genotypes as some of these were clearly grouped in the Haarlem complex and others to genotypes Sierra Leone-1 or -2 (Fig. 2). Overall, there is a very high concordance between genotype classification based on spoligotyping data according to SpolDB4 [13] and to that based on MIRU-VNTR data. However, MIRU-VNTR typing is clearly superior to spoligotyping as it allows a clear genotype identification also for strains of heterogeneous (ill defined) spoligotype clades such as the T-family [13]. Furthermore, compared to spoligotyping that is based on the analysis of one locus only it is more reliable to measure genetic distance since it based on 24 independent loci scattered around the genome [12].

To further investigate the deep phylogenetic branching we investigated sequence polymorphisms within the polymorphic pks1/15 locus. Interestingly, nearly 70% of all strains investigated (n = 66, Table 2) showed a 7 bp deletion that is typical for a major MTBC lineage so called Euro-American lineage according to Gagneux et al. [6]. The wild type pks1/15 sequence was obtained for strains of genotypes Beijing, EAI, and West African-1 while all West African-2 strains had a 6 bp deletion which has also been described before [24]. These data indicate that strains of the Euro-American lineages might be the major cause of TB in West Africa, however, with a large diversification in several well defined phylogenetic lineages as detected by MIRU-VNTR typing (Table 2).

If compared with other studies, it is striking that the *M. tuberculosis *Cameroon lineage, which represents 40% of TB cases in Cameroon [17] and 30% in Burkina Faso [25], was not prevalent in Sierra Leone where it represents only 4% of all strains investigated. This indicates a geographical influence on the distribution of MTBC lineages in West Africa. Possible reasons, e.g. differences in host-pathogen interaction [6], are not yet clear and represent an interesting field for future research.

Due to the limited amount of available patient information and the small study sample, we could not perform a deeper analysis of possible associations between disease characteristics and infection with certain genotypes. However, a striking observation made is that out of 4 Beijing strains three are MDR (Table 2). This is in accordance with higher rates of MDR among Beijing strains reported from several parts of the world especially Eastern Europe [14,26]. Interestingly, the level of MDR was also unequally distributed among strains of other lineages (Table 2). However, due to the small numbers further studies are necessary to investigate this finding.

To analyze the overall discriminatory power of IS*6110 *DNA fingerprinting plus spoligotyping compared to 24 loci MIRU-VNTR typing plus spoligotyping in the study collection (spoligotyping was not considered alone since it is known that its discriminatory power is too low), we have determined the cluster rates obtained with the two different combinations. IS*6110 *DNA fingerprinting plus spoligotyping resulted in 79 distinct typing patterns and grouped 30 strains (31% of all) in 12 different clusters ranging in size from 2 to 5 strains (data not shown). The combination of MIRU-VNTR and spoligotyping had a slightly higher discriminatory power with 85 distinct types reducing the number of clusters to 10 with 22 strains (23% of all).

If the correlation between the two typing schemes is considered, there were a total of eight IS*6110 *DNA fingerprint/spoligotyping clusters that were split by MIRU-VNTR typing (data not shown) with confirmed changes in at least two loci making a real epidemiological linkage rather unlikely [27]. The highest number of MIRU-VNTR differences (12 loci out of 24 had a variation) occurred in a cluster with four EAI genotype strains with just one IS*6110 *band and an identical spoligotype. However, this is not surprising as in this case IS*6110 *DNA has no discriminatory power which can not sufficiently be increased by additional use of just spoligotyping in the case of EAI strains. Out of the 10 MIRU-VNTR/spoligotyping clusters, five were split up by differences in IS*6110 *DNA fingerprint patterns (one up to three bands, data not shown). The combination IS*6110 *DNA fingerprinting with MIRU-VNTR typing yielded the best discrimination of the strains analyzed resulting in 91 different types and only 6 clusters with 12 strains (13%).

These data suggest that the discriminatory power of both typing schemes is not optimal in this high incidence setting. This might be explained by our sampling procedure (short study period/not population based) which made it difficult to detect real transmission chains. However, it is noteworthy that the epidemiological significance of the discordant cases of clustering, especially for those involving slight changes in IS*6110 *DNA fingerprint patterns, remains unknown given the absence of epidemiological and contact tracing data for individual patients. Furthermore, the discriminatory power of molecular typing techniques can be significantly influenced by the composition of the MTBC population investigated. It has already been shown that IS*6110 *DNA fingerprinting is not discriminative in strains with lower band numbers as also observed for strains of the EAI genotype in this study [28]. In a diverse population of MTBC strains such as in Sierra Leone, this might result in a lower correlation between different typing methods if identical molecular typing patterns were considered.

However, the overall concordance between both typing systems is high if similarity rather than identity is considered. The close relationship of strains in IS*6110 *DNA fingerprinting/spoligotyping and MIRU-VNTR typing/spoligotyping cluster, respectively, is clearly supported by the fact that the MIRU-VNTR changes of strains in fingerprint clusters and changes of IS*6110 *DNA banding patterns of strains in MIRU-VNTR clusters are mainly small, respectively.

## Conclusion

Our study confirmed a high level of drug resistance in patients registered for re-treatment in the Western Area and Kenema districts in Sierra Leone. This demonstrates the need for implementing routine drug susceptibility testing for all culture positive cases and improved public health measures to improve the control of the spread of drug resistant and MDR strains in Sierra Leone.

MIRU-VNTR typing proved to be an excellent tool for analyses of the population structure of MTBC strains. It allows the application of automated tools for genotype identification (such as the MIRU-VNTRplus database [23]) and is well suited for the identification of new phylogenetic lineages in a study population. Together with spoligotyping, its discriminatory power is superior to the combination of spoligotyping and IS*6110 *DNA fingerprinting, although the highest discriminatory power was achieved by the combination of all three methods.

The population structure of MTBC strains from Sierra Leone was found to be surprisingly divers with a total of 11 different genotypes/phylogenetic lineages. With 24% of all strains investigated, *M. africanum *still represent a significant number of cases which appears to be not lower compared to that reported from a study performed in years 1992/1993 in Sierra Leone [20]. However, the majority of strains belonged to the *M. tuberculosis *European American lineage [6], which can be further subdivided in seven sub lineages of which two have not been described before. The consequences of this intriguing high MTBC population diversity for the TB epidemic in this high incidence setting as well as for the application of new diagnostic tests or treatment strategies are not yet clear and need to be urgently addressed in further studies.

## Methods

### Study design

All smear positive cases registered for re-treatment (failure at month 5 or above, relapses, treatment after interruption) between March 2003 and June 2004 in the Western Area and Kenema districts in Sierra Leone were recruited. Direct microscopy was performed on all cases at the TB Reference Laboratory, Freetown, Sierra Leone, before shipment to Germany. Sputum samples were shipped to the Supranational Reference Laboratory (SRL) in Borstel, Germany, for cultivation, susceptibility testing, and molecular characterization.

### Strain cultivation and drug susceptibility testing

Primary isolation and cultivation of mycobacterial isolates were performed at the SRL in Borstel as described elsewhere [29]. For all strains, resistance to the key antimycobacterial drugs (INH, RMP; ethambutol, [EMB], streptomycin [SM], pyrazinamide [PZA]) was determined by using the proportion method on Löwenstein-Jensen (LJ) medium. If growth was insufficient, drug-susceptibility testing was performed by using the modified proportion method in BACTEC 460TB according to the manufacturer's instructions (Becton-Dickinson).

### DNA techniques

Extraction of genomic DNA from the mycobacterial strains and DNA fingerprinting using IS*6110 *as a probe were performed according to a standardized protocol as described elsewhere [9]. All isolates were analysed by the spoligotyping technique as described previously by Kamerbeek et al. [10]. For MIRU-VNTR genotyping twenty-four loci were amplified by PCR as described previously [12,27]. Briefly, analyses were performed using multiplex PCRs, Rox-labeled MapMarker 1000 size standard (BioVentures, Inc, Murfreesboro, USA) and the ABI 3100 sequencer with 16 capillaries (Applied Biosystems, Foster City, U.S.A). Sizing of the PCR fragments and assignment of the various VNTR alleles were done using customized GeneScan and Genotyper software packages (Applied Biosystems).

The molecular typing data were analysed with the Bionumerics software (version 4.5; Applied Maths, Sint-Martens-Latem, Belgium) as instructed by the manufacturer. Similarities of IS*6110 *RFLP, spoligotyping and MIRU-VNTR patterns were calculated by using the Dice or the categorical coefficient, respectively. A dendrogram was generated using the unweighted pair group method with arithmetic averages (UPGMA). Minimum Spanning Tree analysis based on MRIU-typing data was done by using the categorical coefficient. Creation of hypothetical types was not allowed. The priority rule was to link types first that had highest number of Single Locus Variants.

Identification of MTBC genotypes was carried out by using the MIRU-VNTR*Plus *database [23].

In addition, all strains were investigated for variation of the pks1/15 gene by direct sequencing of PCR fragments as described previously [6].

## Authors' contributions

SH: Conception and design of the study, acquisition, analysis and interpretation of data, drafting and revising of the article, given final approval to this version to be published. EP: Conception and design of the study, drafting and revising of the article, given final approval to this version to be published. BO: Conception and design of the study, drafting and revising of the article, given final approval to this version to be published. AGG: Conception and design of the study, drafting and revising of the article, given final approval to this version to be published. LW: Conception and design of the study, drafting and revising of the article, given final approval to this version to be published. FD: Conception and design of the study, drafting and revising of the article, given final approval to this version to be published. SR–G: Conception and design of the study, interpretation of data, drafting and revising of the article, given final approval to this version to be published. SN: Conception and design of the study, acquisition, analysis and interpretation of data, drafting and revising of the article, given final approval to this version to be published.

## Supplementary Material

Additional file 1 **Figure S1**. 24 loci MIRU-VNTR typing and spoligotype patterns of the 97 strains investigated. The strains genotypes are ordered in a dendogram based on the similarity of their MIRU-VNTR typing data (D_C _Cavalli-Sforza coefficient, UPGMA). The tree was calculated using the freely assessable MIRU-VNTR*plus *database. MIRU-VNTR loci order: 154 (MIRU 02), 424 (VNTR 42), 577 (VNTR 43), 580 (MIRU 04), 802 (MIRU 40), 960 (MIRU 10), 1644 (MIRU 16), 1955, 2059 (MIRU 20), 2163b (QUB-11b), 2165 (ETRA), 2347 (VNTR 46), 2401 (VNTR 47), 2461 (VNTR 48), 2531 (MIRU 23), 2687 (MIRU 24), 2996 (MIRU 26), 3007 (MIRU 27), 3171 (VNTR 49), 3192 (MIRU 31), 3690 (VNTR 52), 4052 (QUB-26), 4156 (VNTR 53), 4348 (MIRU 39) Click here for file
