# Supplementary figures and images for: High genetic diversity among Mycobacterium tuberculosis complex strains from Sierra Leone
Source: BMC Microbiol. 2008 Jun 25;8:103. doi: 10.1186/1471-2180-8-103 (PMC2447842; doi:10.1186/1471-2180-8-103)

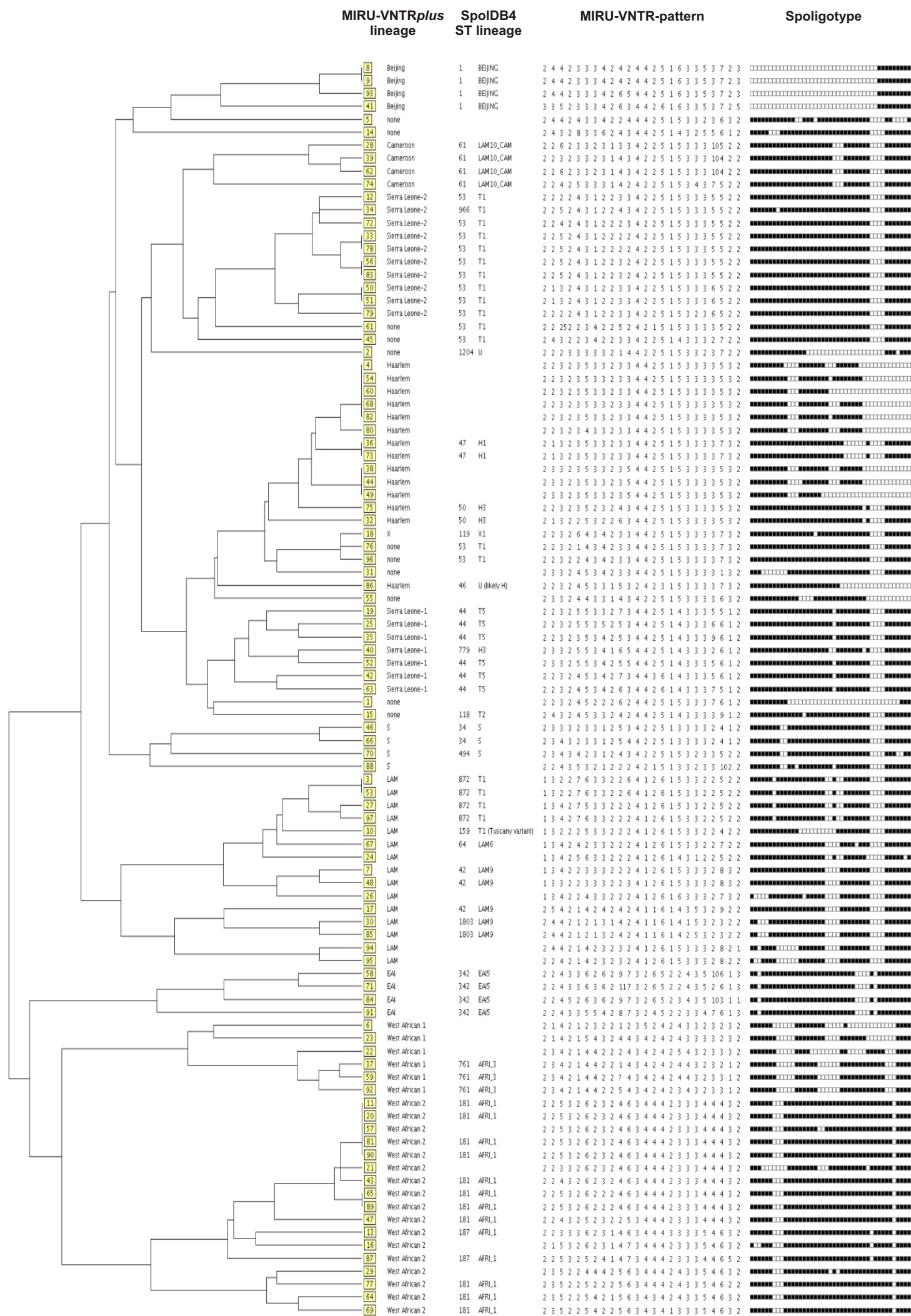

Supplement: Additional file 1 — Figure S1. 24 loci MIRU-VNTR typing and spoligotype patterns of the 97 strains investigated. The strains genotypes are ordered in a dendogram based on the similarity of their MIRU-VNTR typing data (DC Cavalli-Sforza coefficient, UPGMA). The tree was calculated using the freely assessable MIRU-VNTRplus database. MIRU-VNTR loci order: 154 (MIRU 02), 424 (VNTR 42), 577 (VNTR 43), 580 (MIRU 04), 802 (MIRU 40), 960 (MIRU 10), 1644 (MIRU 16), 1955, 2059 (MIRU 20), 2163b (QUB-11b), 2165 (ETRA), 2347 (VNTR 46), 2401 (VNTR 47), 2461 (VNTR 48), 2531 (MIRU 23), 2687 (MIRU 24), 2996 (MIRU 26), 3007 (MIRU 27), 3171 (VNTR 49), 3192 (MIRU 31), 3690 (VNTR 52), 4052 (QUB-26), 4156 (VNTR 53), 4348 (MIRU 39) [file 1471-2180-8-103-S1.pdf]
